# Uso de Estatina Associado ao Treinamento Físico: Uma Combinação Perfeita

**DOI:** 10.36660/abc.20210509

**Published:** 2021-08-09

**Affiliations:** 1 Hospital de Clínicas de Porto Alegre Porto AlegreRS Brasil Hospital de Clínicas de Porto Alegre - Grupo de Vascular e Exercício – VascuEx, Porto Alegre, RS - Brasil; 2 Faculdade Cenecista Santo Ângelo Santo ÂngeloRS Brasil Faculdade Cenecista Santo Ângelo - Curso de Fisioterapia, Santo Ângelo, RS - Brasil; 3 Universidade Federal do Rio Grande do Sul - UFRGS Porto AlegreRS Brasil Universidade Federal do Rio Grande do Sul – UFRGS, Porto Alegre, RS - Brasil

**Keywords:** Dislipidemias/prevenção e controle, Síndrome X Metabólica, Inibidores de Hidroximetilglutaril-CoA Redutases, Fatores de Risco, Exercício, Esportes, Natação

A dislipidemia e a síndrome metabólica crescem exponencialmente nos últimos anos, inclusive em crianças, principalmente pela baixa qualidade da alimentação e pelo elevado nível de sedentarismo populacional.^1^^–^^3^ Essas duas doenças são importantes fatores de risco para o desenvolvimento de doenças cardiovasculares.^4^ A redução da lipoproteína de baixa densidade (LDL), bem como do colesterol total (CT) e da relação CT pela lipoproteína de alta densidade (HDL – CT/HDL) são as principais formas de prevenção cardiometabólica.^5^ O uso de estatinas é considerado tratamento padrão, sendo a medida farmacológica mais eficaz para melhorar o perfil lipídico.^6^ No entanto, existem alternativas não farmacológicas que também são eficazes, sendo uma delas o exercício físico.

Diferentes evidências trazem a importância da adesão ao treinamento físico regular como forma de prevenção primária ou secundária no desenvolvimento ou agravamento de doenças cardiometabólicas.^7^^–^^9^ No entanto, ainda não há um consenso sobre o efeito da combinação entre o uso de estatinas com o treinamento físico, duas poderosas ferramentas que, combinadas, podem promover um impacto ainda maior no perfil lipídico. Foi o que Costa et al.^10^ fizeram nesta edição dos Arquivos Brasileiros de Cardiologia, analisando a influência do uso de sinvastatina nas adaptações lipídicas decorrentes do treinamento aeróbico e de força em meio aquático em mulheres idosas com dislipidemia. Para tal, foram analisadas 69 mulheres idosas (66,13±5,13 anos), sedentárias, dislipidêmicas, tanto não usuárias quanto usuárias de sinvastatina (20 mg e 40 mg).

Vamos destacar os principais pontos metodológicos do artigo a seguir: trata-se de um ensaio clínico randomizado, com 3 grupos (treinamento aeróbico em meio aquático – WA; treinamento de força em meio aquático – WR; grupo controle – GC). A intervenção teve duração de 10 semanas (2 vezes por semana) para todos os grupos. O grupo WA foi composto por 23 mulheres (10 em uso de estatina e 13 sem uso de estatina); o grupo WR foi composto por 23 mulheres (9 em uso de estatina e 14 sem uso de estatina); e o GC foi composto por 23 mulheres (9 sem uso de estatina e 14 sem uso de estatina). Destacamos aqui o primeiro ponto positivo do estudo: randomização bem executada com n amostral igual em todos os grupos e distribuição correta de uso de estatina.

Em relação ao programa de treinamento físico aquático, as participantes dos grupos WA e WR realizaram 2 sessões de familiarização antes do início do seguimento. Além disso, a intensidade de exercício foi aumentada após 5 semanas de intervenção. As sessões de exercício tiveram duração de 45 minutos, sendo 8 minutos de aquecimento, 30 minutos de parte principal e 7 minutos de desaquecimento. Para o grupo WA, adotou-se o treinamento intervalado com intensidade entre 90% e 100% da frequência cardíaca correspondente ao limiar anaeróbio (FC_LA_). Já o grupo WR realizou os exercícios adotando velocidade máxima de execução, e também manteve um tempo fixo de 1 minuto e 20 segundos para cada exercício, enquanto o GC realizou um programa não periodizado de exercícios de relaxamento em imersão, com a finalidade de manter a mesma quantidade de sessões semanais dos outros grupos. O segundo mérito do trabalho foi controlar a intensidade de exercício pela FC_LA_, além de periodizar o treinamento físico, planejando o incremento da intensidade durante o seguimento.

A seguir, destacaremos os principais achados do estudo: em resumo, o uso de estatina associado ao treinamento físico melhora LDL, CT e CT/HDL. Quanto? Participantes medicadas para LDL: −5,58 a 25,18 mg.dl^−1^; CT: −3,41 a 25,89 mg.dl^−1^; CT/HDL: −0,37 a 0,61 mg.dl^−1^, com p significativo quando comparado às participantes não medicadas, sendo essa redução estatisticamente significativa apenas para o grupo WR. De modo geral, o uso de estatina associado ao treinamento de força em meio aquático parece ser a estratégia mais eficaz na redução dos parâmetros lipídicos avaliados no presente estudo.

Sabemos da importância do treinamento físico aeróbico, principalmente pela sua proteção em relação a diversos fatores de risco cardiovascular. No entanto, diferentes evidências sugerem que o treinamento de força é a ferramenta mais eficaz para melhorar o perfil lipídico, principalmente aumentando o HDL, e consequentemente reduzindo CT/HDL.^11^ Durante a prática clínica, os especialistas sabem que o treinamento físico combinado, ou seja, aeróbico e força juntos, na mesma sessão ou em sessões diferentes, apresenta benefícios em diversos parâmetros e acaba sendo o mais utilizado no dia a dia profissional, somando os benefícios tanto do exercício aeróbico quanto do exercício de força. O estudo apresenta muitos méritos, no entanto podemos sugerir que para os próximos estudos, seja comparado o efeito do treinamento combinado e também em homens dislipidêmicos, inclusive ampliando a faixa etária, visto que os resultados do presente estudo servem apenas para mulheres idosas.
