# Supplementary material for: Uso de Estatina Associado ao Treinamento Físico: Uma Combinação Perfeita
Source: Arq Bras Cardiol. 2021 Aug 9;117(2):279–80. [Article in Portuguese] doi: 10.36660/abc.20210509 (PMC8395793; doi:10.36660/abc.20210509)
Supplement: Translation

# Translation: Statin Associated With Physical Training: A Perfect Combination

**Affiliations:** 1 Porto AlegreRS Brazil Hospital de Clínicas de Porto Alegre - Grupo de Vascular e Exercício – VascuEx, Porto Alegre, RS - Brazil; 2 Santo ÂngeloRS Brazil Faculdade Cenecista Santo Ângelo - Curso de Fisioterapia, Santo Ângelo, RS - Brazil; 3 Porto AlegreRS Brazil Universidade Federal do Rio Grande do Sul - UFRGS, Porto Alegre, RS - Brazilx

**Keywords:** Dyslipidemias/prevention and control, Metabolic Syndrome X, Hydroxymethylglutaril-CoA-Reductases, Risk Factors, Exercise, Sports, Swimming

Dyslipidemia and metabolic syndrome have grown exponentially over the recent years, including in children, mainly due to the low quality of food and the high level of a sedentary lifestyle.^1^^–^^3^ These two diseases are important risk factors for the development of cardiovascular diseases.^4^ Reduction of low-density lipoprotein (LDL), as well as total cholesterol (TC) and the TC to high-density lipoprotein ratio (HDL – TC/HDL) are the main forms of cardiometabolic prevention.^5^ The use of statins is considered a standard treatment and the most effective pharmacological measure to improve the lipid profile.^6^ However, there are non-pharmacological alternatives that are also effective, one of which is physical exercise.

There is evidence on the importance of regular physical training as a form of primary or secondary prevention in the development or worsening of cardiometabolic diseases.^7^^–^^9^ However, there is no consensus on the effect of combining statins with physical training, two powerful tools that, combined, can promote an even greater impact on the lipid profile. This is what the Costa et al.^10^ did in this edition of *Arquivos Brasileiros de Cardiologia* — they analyzed the influence of simvastatin on lipid adaptations resulting from aerobic and strength water training in elderly women with dyslipidemia. To this end, 69 female elderly sedentary dyslipidemic non-users and users of simvastatin (20 mg and 40 mg) (66.13±5.13 years) were analyzed.

Below are the main methodological points of the article: it is a randomized clinical trial with 3 groups (aerobic water training – WA; strength water training – WR; control group – CG). The intervention lasted 10 weeks (2 times a week) for all groups. The WA group included 23 women (10 using statin and 13 not using statin); the WR group consisted of 23 women (9 using statin and 14 not using statin); and the CG consisted of 23 women (9 not using statin and 14 not using statin). This is the first positive point of the study: well-executed randomization with equal sample n in all groups and correct distribution of statin use.

Regarding the water training program, participants of the WA and WR groups attended 2 induction sessions before the beginning of follow-up. Besides, exercise intensity was increased after 5 weeks of intervention. The exercise sessions lasted 45 minutes, including 8 minutes of warm-up, 30 minutes of exercise and 7 minutes of cool-down. For the WA group, interval training was adopted with an intensity of 90% to 100% of the heart rate corresponding to the anaerobic threshold (HR_AT_). The WR group performed the exercises adopting a maximum execution speed, and maintained a fixed duration of 1 minute and 20 seconds for each exercise, while the CG performed a non-periodized program of immersion relaxation exercises in order to maintain the HART same number of weekly sessions as the other groups. The second merit of the study was to control exercise intensity by HR_AT_, in addition to periodizing physical training, planning an increased intensity during follow-up.

Below are the main findings of the study: in summary, statin use associated with exercise training improves LDL, TC and TC/HDL. How much? Participants medicated for LDL: −5.58 to 25.18 mg.dl^−1^; TC: −3.41 to 25.89 mg.dl^−1^; TC/HDL: −0.37 to 0.61 mg.dl^−1^, with significant p compared to non-medicated participants. This reduction is only statistically significant for the WR group. In general, the use of statins associated with strength water training seems to be the most effective strategy in reducing the lipid parameters evaluated in the study.

We are aware of the importance of aerobic physical training, especially for its protection against multiple cardiovascular risk factors. However, there is evidence suggesting that strength training is the most effective tool to improve the lipid profile, especially increasing HDL, and consequently reducing TC/HDL.^11^ During clinical practice, specialists know that combined physical training, that is, aerobic and strength training together, in the same session or in different sessions, has benefits in several parameters and turns out to be the training style most used in professional daily life, adding up the benefits of aerobic exercise and strength exercise. The study has many merits, but we suggest future studies comparing the effect of combined training and studies on dyslipidemic men, including an expanded age range, as these study findings only apply to elderly women.
